# Surgery for knee osteoarthritis in younger patients: A Swedish Register Study

**DOI:** 10.3109/17453670903413186

**Published:** 2010-04-06

**Authors:** Annette W-Dahl, Otto Robertsson, Lars Lidgren

**Affiliations:** Department of Orthopedics, Clinical Sciences Lund, Lund University Hospital, LundSweden

## Abstract

**Background and purpose** In Sweden, surgery for knee osteoarthritis (OA) in patients younger than 55 years of age has doubled during the last 10 years. We evaluated the use of 3 surgical alternatives: high tibial osteotomy (HTO), unicompartmental arthroplasty (UKA), and total knee arthroplasty (TKA). We also examined the outcome, expressed by rate of revision.

**Methods** The numbers of all procedures during 1998–2007 were obtained from the Swedish Knee Arthroplasty Register (SKAR) (UKA < 55 years: n = 1,050; UKA ≥ 55 years: n = 7,743; TKA < 55 years: n = 2,832; TKA ≥ 55 years: n = 62,829) and the National Board of Health and Welfare (NHW) (HTO 25–55 years: n = 2,266). The revision rate (presented as life tables) was based on the SKAR material for arthroplasties. For HTOs, a single institutional series of 450 patients aged 30–64 years was used to calculate the revision rate and to compare it to that for UKAs (n = 4,799; age 30–64 years).

**Results** During the 10 years, the use of TKA in patients younger than 55 years increased fivefold. While UKA increased threefold, its use diminished in the last 2 years. Although the use of HTO halved during the period, it is still used more often than UKA. The risk of revision increased in patients younger than 55 years and was lower for TKA (9%) than for UKA (24%). The revision rate was similar for HTO (17%) and for UKA (17%) in patients aged 30–64 years.

**Interpretation** TKA is the preferred method for young OA patients in Sweden today. The use of HTO and UKA has diminished, and as the few operations are spread over many hospitals, there is a risk of gradual loss of experience with respect to patient selection and surgical routine—with a negative effect on outcome. Thus, there is a risk that these treatment alternatives for younger patients will eventually be abandoned.

## Introduction

During the past 10 years, knee surgery for OA (i.e. high tibial osteotomy and arthroplasty) in patients less than 55 years of age has doubled in Sweden (Swedish Knee Arthroplasty Register 2008, the Swedish National Board of Health and Welfare 2008). There are several plausible reasons for this. The incidence of knee OA may have increased in the younger population, their grade of OA may have become more severe, or there may have been a broadening of the indications for surgery as the surgeons have gained confidence in the surgical treatment.

In the Swedish guidelines for treatment of knee OA (The Medical Product Agency 2004) as well as in international guidelines, information, exercise, and weight control constitute the basis of the OA treatment that should be offered to all OA patients (the Medical Product Agency 2004, Zhang et al. 2007). In later stages of the disease, surgical treatment becomes the main alternative.

The Swedish guidelines give no recommendations regarding the choice of surgical methods. UKA was used for most patients when the Swedish knee arthroplasty register started in 1975. However, with the arrival of TKA, the new type quickly succeeded the use of UKA. Still, there are those who favor HTO and UKA in selected cases, claiming that the surgery is less extensive, bone sparing, allows a higher degree of physical activity, and has the option of being converted to TKA later on.

One reason for the decreasing popularity of these latter treatment alternatives may be that they have been considered to be somewhat difficult to perform technically, which has been supported by a study showing that the revision rate of UKA is affected by operating volume (Robertsson et al. 2001). While UKA has been used for unicompartmental disease in patients of all ages, the HTO has mainly been offered to those who are younger and more physically active.

The purpose of this study was to examine the use of different surgical options for knee OA in Sweden and their revision rates in patients less than 55 years of age, from 1998 through 2007.

## Methods

The numbers of UKA and TKA procedures in patients operated on for knee OA between 1998 and 2007 inclusive were obtained from the Swedish Knee Arthroplasty Register (SKAR). The numbers are presented per hospital.

The number of HTOs was gathered from the Swedish National Board of Health and Welfare (NHW) statistics on inpatient procedures, which did not include the diagnosis leading to surgery. We searched for patients who were 25–55 years of age and who had an operation with the surgical code NGK59 (angulation, rotation, or displacement osteotomy of knee or lower leg). Thus, the numbers may include some patients who had types of osteotomy other than HTO and diagnoses other than knee OA. Furthermore, patients who might have had outpatient surgery were not included. As outpatient osteotomy is rare, and as the number of osteotomies for limb deformities is known to be rather small, we feel confident that most of the procedures accounted for were HTOs in patients with OA.

The numbers of HTOs are presented per county. Sweden is divided into 21 Ccounties, and each county has the responsibility for public healthcare.

When assessing the cumulative revision rate (CRR), we used national data for UKA and TKA from the SKAR with revision being defined as a second operation during which one or more components were exchanged, removed, or added (including arthrodeses and amputations).

With respect to the outcome of HTO, no national data were available. Instead, we studied 450 HTOs performed during 1993–2005 at Lund University Hospital, Sweden. All the osteotomies were performed using the hemicallotasis technique. The patients included were 30–64 years old at surgery instead of < 55 years, in order to get an adequate number of procedures to analyze. Revision was defined as a second operation with a re-osteotomy or an arthroplasty. For comparison, we obtained national results from the SKAR of UKAs in patients with OA who were in the same age group at surgery (30–64 years old).

### Statistics

When plotting the curves, the cumulative revision rate was calculated by the life table method using 1-month intervals and the confidence intervals (CIs) using the Wilson quadratic equation with Greenwood and Peto effective sample-size estimates. Curves were cut off when 40 knees remained at risk. The praxis of cutting curves at the right when 40 procedures remain at risk is historical, but has practical benefits. When less than 40 are left at risk, one or few revisions have a large effect on the final CRR. Although the confidence interval also widens, this is not always well understood by those not used to interpreting such curves. The practical benefit is that it becomes easier to produce figures of standardized size, as large fluctuations in the confidence interval—at the end of the curve—disappear.

When comparing groups, the log-rank test was used.

## Results

**Figure 1.**
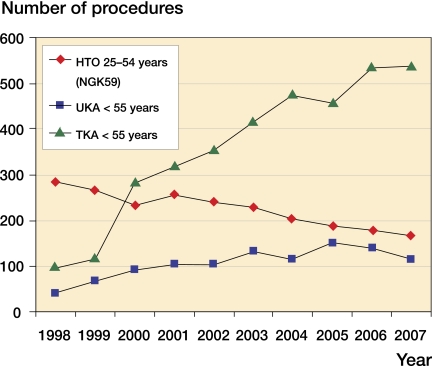
The numbers of HTOs (NGK59), UKAs and TKAs performed in patients younger than 55 years of age in Sweden, 1998–2007 (NHW 2008; SKAR 2008).

In 2000, the use of TKA in younger patients started to increase and this continued so that in 2007 the number had become 5 times that in 1998. For UKA, the use in younger patients (< 55 years of age) tripled during 1998–2005 but then it decreased over the next 2 years. With regard to HTO, this was the most common surgical alternative for knee OA in young patients until 2000. However, the number has halved since 1998, although HTO still is more common than UKA (Figure 1).

**Figure 2.**
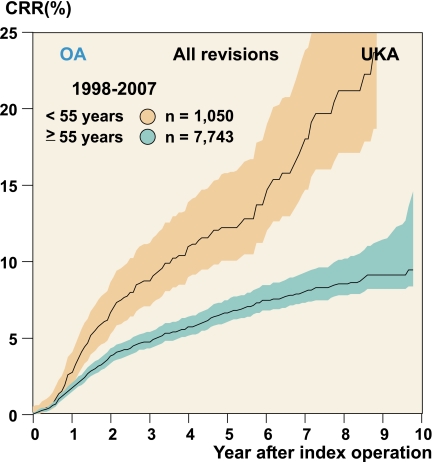
Revision rate for UKA.

The risk of revision of primary UKA decreased with age. For the age group less than 55 years of age, the 10-year CRR was almost 24%, which was 3 times higher than for patients who were 55 years and older (Figure 2).

**Figure 3.**
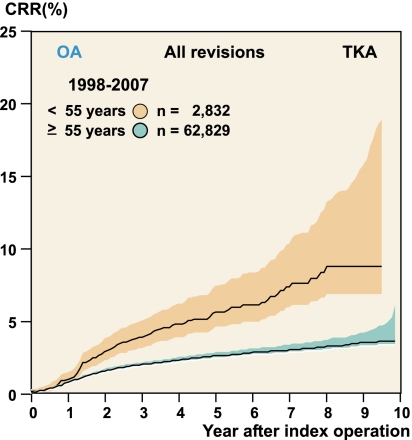
Revision rate for TKA.

For TKA, the pattern was the same as for UKA with increased risk of revision with younger age. However, the CRR was lower in TKA than in UKA, with a 10-year CRR of 9% in those patients less than 55 years of age (Figure 3).

**Figure 4.**
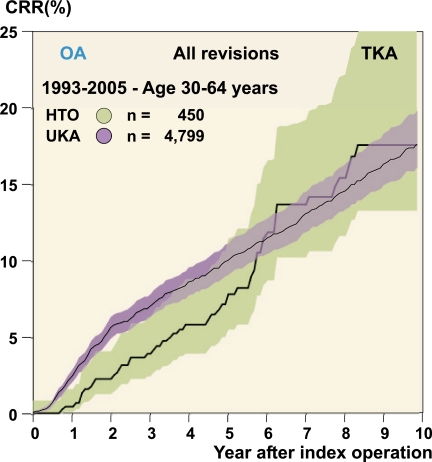
Revision rate for the Lund cohort of HTO and UKA patients; data from the Swedish Knee Arthroplasty Register.

For the Lund cohort of HTOs, the 10-year CRR was 17% at 10 years. In comparison, based on the national data from the SKAR, the CRR for UKA patients 30–64 years of age was quite similar at 10 years although the risk of revision for the HTOs appeared to be somewhat lower for the first years (Figure 4).

In 2007, one-third of the UKA patients were operated at hospitals that performed 15 UKAs or less per year. In 1997, when UKA was used somewhat more frequent, the situation was the same (Table). 39% of the HTO patients who were operated on in 2007 had surgery in counties that performed 15 or less HTOs per year (Table).

**Table:** Numbers of UKAs, HTOs, and TKAs in 1998 and 2007 presented as numbers of hospitals or regions

	UKA		HTO		TKA		
No. of procedures	1998 (63 hospitals)	2007 (60 hospitals)	1998 (21 regions)	2007 (21 regions)	No. of procedures	1998 (76 hospitals)	2007 (76 hospitals)
< 5	21	16	2	7	< 50	34	10
5–10	6	21	6	6	50–100	32	21
11–15	11	7	6	3	101–150	7	20
16–20	6	2	1	3	151–200	2	11
21–25	2	7	2	0	201–250	0	6
26–30	7	1	0	0	251–300	1	2
> 30	8	6	4	2	> 300	0	5

In 2007, 43% of the TKA patients were operated at hospitals performing 150 or less TKA per year (Table).

## Discussion

In the treatment of young patients with OA of the knee, the increase in knee reconstructions not only applies to Sweden. In the Australian Joint Replacement Register (AOANJRR), the number of knee reconstructions in patients less than 55 years of age increased by 40% from the start of the register in 2002 until 2007 (Australian Orthopedic Association National Joint Replacement Registry 2008).

The information regarding HTO is less certain due to the lack of national HTO registers, and this is a limitation of our study. We used a consecutive series from one Swedish hospital to estimate the revision rate for HTO, which may not have been representative of the national outcome. Still, our HTO cohort is the largest in Sweden. The reason that we analyzed the results for patients less than 65 years of age instead of those aged less than 55 was in order to obtain an adequate sample size.

As younger patients have a longer expected lifetime and higher activity levels, the demands on the operated knee are considerably greater than in the older patients. At present, only 8% of the patients who undergo HTO or knee arthroplasty are under 55 years of age. However, considering their higher revision rate and the increasing use of knee arthroplasty, the burden of revision arthroplasty is likely to increase substantially in the future.

Before 2000, HTO was the most common surgical treatment for OA in younger patients (< 55 years) in Sweden. The large increase in the use of knee arthroplasties for this group in 2000 coincided with the time when knee arthroplasty surgery became industrialized in Sweden, by introduction of high-volume units and guarantee of care within 3 months for patients. The trend of offering knee arthroplasty instead of HTO may reflect a change in opinion regarding the treatment of choice. However, it may also be affected by the fact that volume of arthroplasty surgery has become an outcome measure of hospital output as well as a source of income, and that the demands on early postoperative care are higher after HTO than after arthroplasty.

Robertsson et al. (2001) showed that hospitals that performed less than 23 UKAs per year had a 1.6 times higher revision rate than units that operated 23 or more. Thus, the routine in patient selection and surgical skill was considered to affect outcome in UKA (Robertsson and Lidgren 2008) and it is highly likely that this also applies to HTO.

In Sweden at present, almost 50% of the UKA operations are performed at hospitals doing less than 23 UKAs per year, and for HTO the volumes are even lower.

As the numbers of HTOs and UKAs are diminishing, and the surgeries are spread over many hospitals, there is a risk of competence being lost. This might adversely affect outcome, and it may ultimately mean that these treatment alternatives will be abandoned. One strategy might be to centralize HTO, UKA, and also surgery in younger patients to a smaller number of surgeons with a special interest and sufficient throughput, with the purpose of maintaining competence and probably improving outcome.
